# Alterations of dynamic and static brain functional activities and integration in stroke patients

**DOI:** 10.3389/fnins.2023.1228645

**Published:** 2023-10-27

**Authors:** Li Guo, Zixuan Zhao, Xu Yang, Weiyang Shi, Peng Wang, Dongdong Qin, Jiaojian Wang, Yong Yin

**Affiliations:** ^1^Graduate School of Kunming Medical University, Kunming, China; ^2^Department of Rehabilitation Medicine, The Affiliated Hospital of Yunnan University, Kunming, China; ^3^Brainnetome Center, Institute of Automation, Chinese Academy of Sciences, Beijing, China; ^4^Department of Radiology, The Affiliated Hospital of Yunnan University, Kunming, China; ^5^Key Laboratory of Traditional Chinese Medicine for Prevention and Treatment of Neuropsychiatric Diseases, Yunnan University of Chinese Medicine, Kunming, China; ^6^Yunnan Key Laboratory of Primate Biomedicine Research, Institute of Primate Translational Medicine, Kunming University of Science and Technology, Kunming, China

**Keywords:** stroke, dynamic amplitude of low-frequency fluctuation, static amplitude of low-frequency fluctuation, dynamic degree centrality, static degree centrality

## Abstract

**Objective:**

The study aimed to investigate the comprehensive characteristics of brain functional activity and integration in patients with subcortical stroke using dynamic and static analysis methods and to examine whether alterations in brain functional activity and integration were associated with clinical symptoms of patients.

**Methods:**

Dynamic amplitude of low-frequency fluctuation (dALFF), static amplitude of low-frequency fluctuation (sALFF), dynamic degree centrality (dDC), and static degree centrality (sDC) were calculated for 19 patients with right subcortical stroke, 16 patients with left subcortical stroke, and 25 healthy controls (HC). Furthermore, correlation analysis was performed to investigate the relationships between changes in brain functional measurements of patients and clinical variables.

**Results:**

Group comparison results showed that significantly decreased dALFF in the left angular (ANG_L) and right inferior parietal gyrus (IPG_R), decreased sALFF in the left precuneus (PCUN_L), and decreased sDC in the left crus II of cerebellar hemisphere (CERCRU2_L) and IPG_R, while significantly increased sDC in the right lobule X of cerebellar hemisphere (CER10_R) were detected in patients with right subcortical stroke relative to HC. Patients with left subcortical stroke showed significantly decreased sALFF in the left precuneus (PCUN_L) but increased sDC in the right hippocampus (HIP_R) compared with HC. Additionally, the altered sDC values in the CER10_R of patients with right subcortical stroke and in the HIP_R of patients with left subcortical stroke were associated with the severity of stroke and lower extremities motor function. A correlation was also found between the altered sALFF values in the PCUN_L of patients with left subcortical stroke and lower extremities motor function.

**Conclusion:**

These findings suggest that time-varying brain activity analysis may supply complementary information for static brain activity analysis. Dynamic and static brain functional activity and integration analysis may contribute to a more comprehensive understanding of the underlying neuropathology of dysfunction in stroke patients.

## Introduction

Stroke is a neurological disease with high incidence and is the second leading cause of death and disability in the world (Krishnamurthi et al., 2020). Motor impairment is the major reason for post-stroke disability. Meanwhile, cognitive deficit negatively impacts the rehabilitation therapy and recovery of stroke patients. Magnetic resonance imaging (MRI) is becoming the most popular neuroimaging technique owing to its high-resolution imaging, non-invasive, as well as its value in identifying imaging biomarkers of neurological and mental diseases (Feng et al., 2015; Tozzi et al., 2020; Lee et al., 2022; Bruin et al., 2023). Based on blood-oxygen-level dependent functional MRI (BOLD-fMRI), the signal strength of spontaneous brain activity within the frequency range of 0.01–0.01 Hz was measured as an amplitude of low-frequency fluctuation (ALFF), which has been used to explore intrinsic neural activity changes in stroke patients (Zhu et al., 2015; Hu et al., 2021; Quan et al., 2022). Meanwhile, graph theory-based network analysis has become a powerful method for exploring brain connectivity of complex brain networks (Bassett and Bullmore, 2017; Bournonville et al., 2018; Sporns, 2018; Shi et al., 2021; Cai et al., 2023). Degree centrality (DC) is an important graph theoretical metric that characterizes the functional integration of a specific voxel. Researchers have identified DC changes in brain regions after stroke (Liu et al., 2019; Yao et al., 2020). Though ALFF and DC have been intensively used to examine alterations of brain functional activity and integration in patients with stroke, the aforementioned studies are static analyses that assumed that the brain neural signals are stationary during fMRI data acquisition without exploring the characteristics of dynamic alteration of brain spontaneous activity over time.

It has been well documented that the brain is a dynamic system. In recent years, an increasing number of researchers have focused on exploring time-varying intrinsic brain activity or functional connectivity (Allen et al., 2014; Calhoun et al., 2014; Liu et al., 2017; Ma et al., 2021; Dong et al., 2022; Xue et al., 2022). The sliding window method is effective and sensitive in exploring temporal variability of brain activity and functional connectivity over the whole brain (Liu et al., 2017; Ma et al., 2021; Dong et al., 2022). Several studies have adopted the sliding window approach to observe functional connectivity or brain activity dynamics in patients with stroke and have found significant changes in dynamic functional connectivity of brain networks (Chen et al., 2018; Bonkhoff et al., 2020, 2021; Bruyn et al., 2023) or dynamic ALFF (dALFF) or dynamic regional homogeneity of brain regions (Chen et al., 2019; Tian et al., 2022; Xie et al., 2022; Chen and Li, 2023). The study of Xie et al. also showed both altered dALFF and static ALFF (sALFF) were associated with the clinical characteristics of patients with stroke (Xie et al., 2022), suggesting that dynamic and static analysis play an important role in understanding the neuropathology of dysfunction in stroke patients. However, limited research has been conducted to comprehensively examine brain functional activity and integration in stroke patients using dynamic and static analysis methods.

Therefore, the major aim of the present study was to explore the comprehensive characteristics of brain functional activity and integration in patients with subcortical stroke by calculating dALFF, sALFF, dynamic DC (dDC), and static DC (sDC). Stroke patients were divided into the right subcortical stroke group and left subcortical stroke group, and the differences in brain metrics between the two groups of patients and healthy control groups (HC) were compared to investigate the pattern of specific changes in brain function caused by different hemisphere lesions. We further evaluated the relationships between neuroimaging measurement changes in the two groups of patients and the severity of stroke, cognitive impairment, and upper and lower extremities motor deficits to investigate whether dysfunctions in stroke patients were associated with changes in brain function. We sought to explore whether dynamic analysis shows similar or complementary information to static analysis. These results may contribute to a more comprehensive understanding of the underlying neuropathology of dysfunction in stroke patients.

## Materials and methods

### Participants and clinical assessments

A total of 38 patients with subcortical stroke (21 with right subcortical stroke and 17 with left subcortical stroke) were recruited from the Department of Rehabilitation Medicine, the Affiliated Hospital of Yunnan University (Kunming, China). The inclusion criteria were as follows: (1) the first-onset stroke (ischemic or hemorrhagic stroke confirmed by computed tomography or MRI), (2) course of stroke between 2 weeks and 3 months, (3) right-handedness before stroke, (4) people aged 18 to 75 years, and (5) manifesting motor or cognitive deficit evaluated by clinical scales. The exclusion criteria were as follows: (1) a history of other neurological or psychiatric disorders and (2) any contraindications for MRI scanning, such as intracranial metal clips, cardiac pacemakers, defibrillators, or aneurysm clips. The severity of stroke, cognitive function, and upper and lower extremities motor functions of all patients were assessed on the same day as MRI scan acquisition by the National Institutes of Health Stroke Scale (NIHSS), the Chinese version of Montreal Cognitive Assessment Basic (MoCA-BC), and the Fugl–Meyer Upper and Lower Extremities assessment (FMA-UE, FMA-LE), respectively. The NIHSS assesses the level of consciousness, eye movements, integrity of visual fields, facial movements, upper and lower limb muscle strength, sensory function, body coordination, language, speech, and neglect of patients (Kwah and Diong, 2014). The cumulative score of all items ranges from 0 to 42 points. A lower score indicates a less severe stroke. The MoCA-BC examines the following domains: executive function, language, orientation, calculation, conceptual thinking, memory, visual perception, attention, and concentration (Chen et al., 2016). The total cumulative score ranges from 0 to 30 points. The higher the score, the better the patient’s cognitive function. The motor domain of FMA focuses on reflex action, movement, and coordination of upper and lower extremities (Gladstone et al., 2002). The total score of the upper extremities (FMA-UE) is 66 points, and the total score of the lower extremities (FMA-LE) is 34 points. The higher the score, the better the residual motor function of patients. Twenty-five gender- and age- matched healthy subjects were recruited as HC; they were right-handed, had no history of other neurological or psychiatric disorders, and had no contraindications for MRI scanning. Meanwhile, the MoCA-BC scores were also obtained from HC. This study was approved by the Medical Research Ethics Committee of the Affiliated Hospital of Yunnan University (No.2022057), and written informed consent was provided and obtained from all participants.

### MRI data acquisition

All participants were examined on the Philips Achieva 3.0 T MR scanner (Philips Healthcare, the Netherlands) with the 32-channel head–neck coil. High-resolution 3D T1-weighted images were acquired with the following parameters: repetition time (TR) =7.5 ms, echo time (TE) = 3.5 ms, flip angle (FA) = 8°, field of view (FOV) = 250 × 250 mm^2^, matrix = 228 × 228, and voxel = 1.1 × 1.1 × 1.2 mm^3^, no gap. The parameters for obtaining resting-state BOLD-fMRI data were as follows: TR = 2,000 ms, TE = 30 ms, FA = 90°, FOV = 240 × 240 mm^2^, matrix = 64 × 64, voxel = 3.75 × 3.75 × 4 mm^3^, no gap, 200 volumes, and the entire scanning procedure lasted for 6 min and 46 s. T2-weighted images were also obtained to identify the location of stroke lesions: TR = 3,500 ms, TE = 108 ms, FA = 90°, FOV = 230 × 230 mm^2^, matrix = 384 × 384, voxel = 0.6 × 0.6 × 6 mm^3^, no gap. The whole MRI scanning process was completed in 13 min and 9 s. All participants were instructed to keep their heads motionless, close their eyes, not think about anything, and avoid falling asleep during the scanning. Additionally, comfortable foam padding and earplugs were used to reduce head motion and scanner noise.

### Lesion mapping

**Figure 1:**
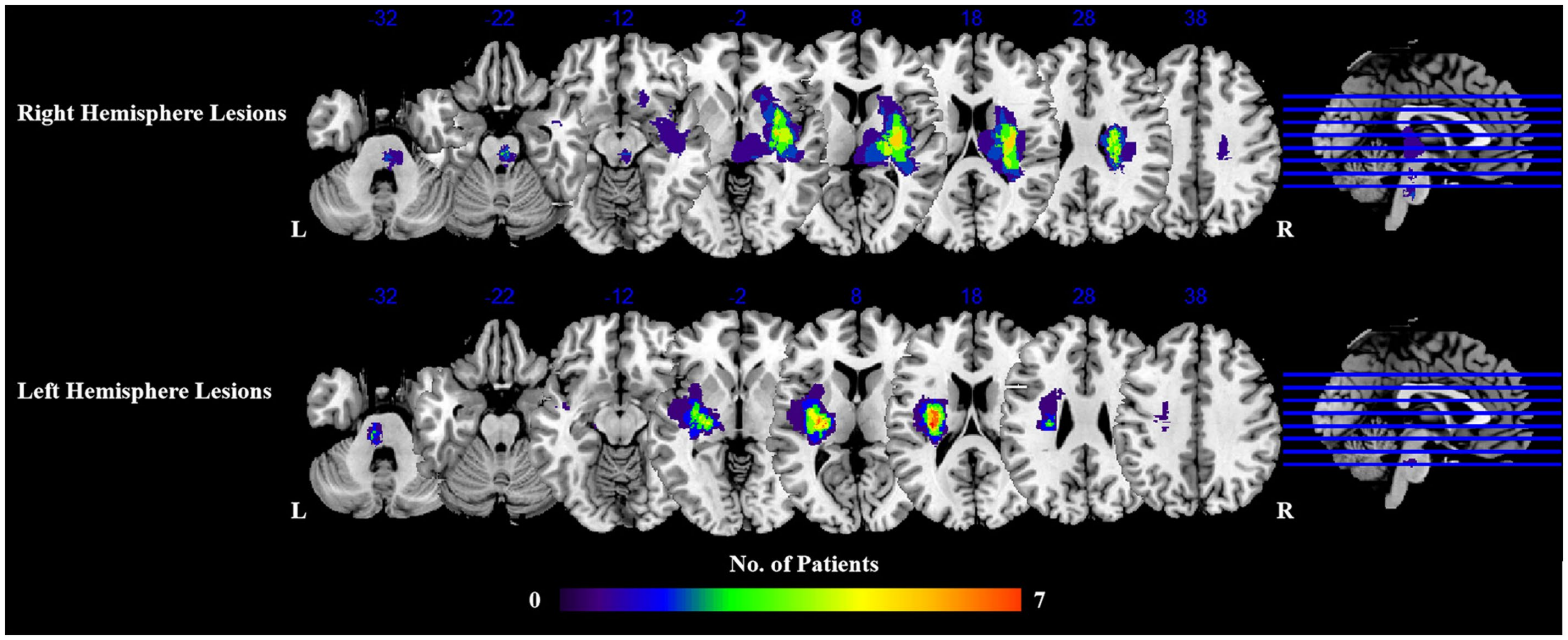
Distribution of lesions of stroke patients. L, left; R, right.

Lesion masks of all patients were first manually delineated on the native 3D T1-weighted MRI images using the software ITK-SNAP (Yushkevich et al., 2006).^1^ Then, the lesion mask of each patient was spatially normalized to a standard brain template (Montreal Neurological Institute (MNI) space). Finally, in order to obtain the overlap map of all lesions, the lesion masks of all right hemisphere stroke and left hemisphere stroke were overlapped, respectively. Figure 1 shows the distribution of lesions in two groups.

### BOLD-fMRI data preprocessing

Resting-state BOLD-fMRI data were preprocessed with the Data Processing and Analysis of Brain Imaging toolkit (DPABI version 6.1; Yan et al., 2016) running on Matlab R2021b with the following steps: (a) the first 10 volumes were removed to stabilize the MRI signal; (b) the remaining 190 volumes were for head motion correction, the mean framewise-displacement (mean FD) was calculated for each participant; (c) spatial normalization to the standard MNI EPI template and each voxel was resampled to 3 × 3 × 3 mm^3^; (d) detrending; (e) Friston-24 head motion parameters, white matter, cerebrospinal fluid, and whole brain signals were regressed out; (f) filtered with a temporal bandpass of 0.01–0.1 Hz; (g) scrubbing to remove the “bad” time points and their 1 forward and 2 back time points on the basis of FD threshold of 0.5 mm. Three patients with maximal head motion that exceeded 2.5 mm displacement or 2.5^°^ of rotation were excluded after image preprocessing. Nineteen patients with right subcortical stroke and 16 patients with left subcortical stroke were left to calculate brain functional measurements.

### Dynamic and static functional activity and integration analysis

The temporal variability of functional measurements of each participant was calculated using temporal dynamic analysis toolkits on DPABI. ALFF was evaluated within the frequency range of 0.01–0.1 Hz. DC calculates the temporal correlation between a voxel and all other voxels. In this study, we obtained DC with Pearson’s correlation coefficients, and the correlation threshold was set at 0.25. According to previous studies, the sliding window length of 50 TRs (100 s) is appropriate to capture dynamic brain activity (Liao et al., 2019; Dong et al., 2022). Therefore, we selected 50 TRs (100 s) as sliding window length and 5 TRs (10 s) as step size to obtain a total of 29 maps of ALFF and DC. Then, the standard deviation of 29 consecutive ALFF and DC values was calculated to get dynamic ALFF and DC maps. The same procedures were performed for each voxel to obtain the whole brain dALFF and dDC maps. DC can be calculated as weight DC or binary DC. In the present study, the positive weighted sum standard deviation DC maps were used for further statistical analysis. The sALFF and sDC were also obtained to identify whether dynamic measurements are similar or different from static measurements to help us understand the neuropathological mechanisms after stroke. Finally, the z-score sALFF and sDC maps, standard deviation ALFF maps, and positive weighted sum standard deviation DC maps were smoothed with a Gaussian kernel of full-width at half-maximum of 8 mm before statistical analysis.

### Statistical analysis

The demographic characteristics of the stroke patient group and HC group were statistically analyzed using Graphpad Prism (software version 9.5.1).^2^ First, the clinical continuous variables data of the stroke patient group and HC group were tested for normal distribution with the Shapiro–Wilk test. Normally distributed variables were reported as mean ± standard deviation, while non-normally distributed variables were shown as median with interquartile range. Then, the group differences in age, education level, and cognitive function were obtained by the two-sample *t*-test or Wilcoxon rank sum test. The chi-square test was performed to detect the sex differences. A *value of p* of <0.05 was considered to be statistically significant. In terms of the group differences in imaging indices, the two-sample t-test was performed with the age, gender, education level, and head motion parameters of the mean FD values as covariates. AlphaSim correction was employed for multiple testing (voxel-wise *p* < 0.005, cluster-wise *p* < 0.05, two-tailed) and the automated anatomical labeling 3 (AAL3) template was applied as the brain mask to obtain brain region with significant differences in each brain functional measurement between the stroke patient group and HC group. Brain regions were excluded if they overlapped with the patient’s lesions to avoid the influence of lesions.

### Correlation analysis

To better investigate the associations between brain functional measurements and clinical symptoms of patients, the mean brain functional measurements within each brain region showing significant differences between the stroke patient group and HC group were calculated. All functional measurements of the two groups of stroke patients were tested for normal distribution by the Shapiro–Wilk test. Then, Pearson correlation analysis or Spearman correlation analysis was used to determine the relationships between brain functional measurements and NIHSS, MoCA-BC, FMA-UE, and FMA-LE scores of the two groups of stroke patients. The significance level was set at a *value of p* of <0.05 with false discovery rate (FDR) correction.

## Results

### Demographic characteristics

Three stroke patients were excluded from further analysis owing to excessive head motion after the resting-state BOLD fMRI data head motion check. The demographic characteristics for the final 35 stroke patients and 25 HC are summarized in Table 1. There are no significant differences in age, education level, and gender between stroke patients and HC (*p* > 0.05). Significantly decreased MoCA-BC scores were found in stroke patients compared to HC (*p* < 0.0001).

**Table 1:** Demographic characteristics of stroke patients and health controls.

	Right subcortical stroke (*n* = 19)	Left subcortical stroke (*n* = 16)	HC (*n* = 25)	Value of *p*
Age (year)	56.11 ± 10.50	57.69 ± 9.43	52.16 ± 8.15	*p*_1_ = 0.1677^a^
				*p*_2_ = 0.0534^a^
Education level (year)	12 (7)	9 (6)	9 (3)	*p*_1_ = 0.3931^b^
				*p*_2_ = 0.7058^b^
Gender (male/female)	10/9	11/5	14/11	*p*_1_ = 0.414^c^
				*p*_2_ = 0.824^c^
MoCA-BC scores	26.00 (6.00)	26.5 (3.75)	29.00 (2.00)	*p*_1_ < 0.0001^b^
				*p*_2_ < 0.0001^b^
**Stroke type**				
Hemorrhagic stroke	9	4		
Ischemic stroke	10	12		
**Location of lesion**				
Corona radiate	2	1		
Basal ganglia	9	6		
Corona radiate and basal ganglia	1	5		
Thalamus	2	0		
Pons	5	4		
Time post-stroke (day)	21.00 (12.00)	17.56 (25.75)		
NIHSS scores	4.90 ± 3.09	3.50 ± 2.13		
FMA-UE scores	38.00 (45.00)	25.25 (36.62)		
FMA-LE scores	19.74 ± 7.00	22.88 ± 6.23		

HC, healthy controls; MoCA-BC, the Chinese version of Montreal Cognitive Assessment Basic; NHISS, National Institute of Health Stroke Scale; FMA-UE, Fugl–Meyer Upper Extremities assessment; FMA-LE, Fugl–Meyer Lower Extremities assessment; p_1_ indicates comparison between the right subcortical stroke group and HC group; p_2_ indicates comparison between the left subcortical stroke group and HC group. ^a^represents value of *p* obtained by the two-sample *t*-test; ^b^represents value of *p* obtained by the Wilcoxon rank sum test; ^c^represents value of *p* obtained by the chi-square test.

### Altered brain functional measurements in stroke patients

**Figure 2:**
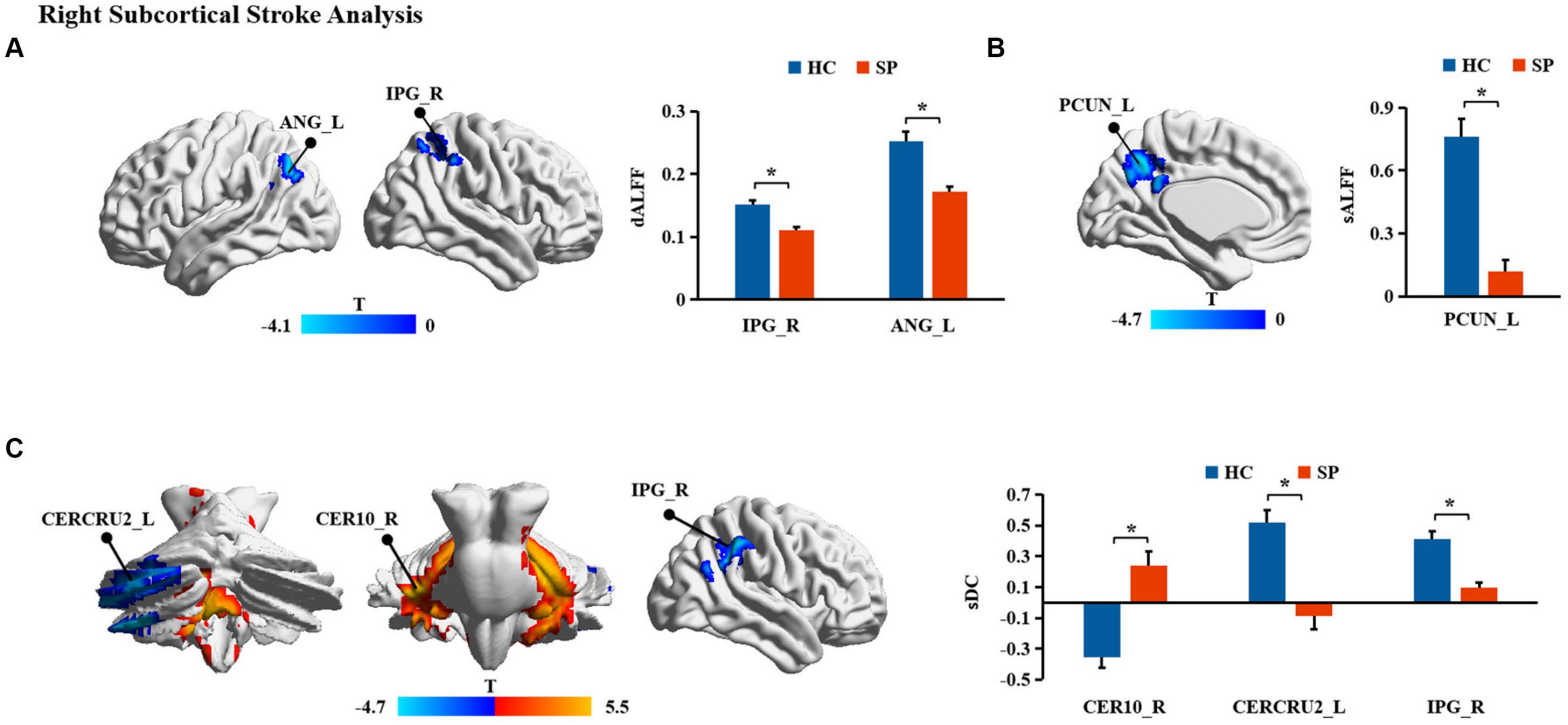
**(A-C)** Brain regions with significant differences in functional measurements between patients with right subcortical stroke compared with health controls (HC). SP, stroke patients; dALFF, dynamic amplitude of low-frequency fluctuation; sALFF, static amplitude of low-frequency fluctuation; sDC, static degree centrality; ANG_L, left angular; IPG_R, right inferior parietal gyrus; PCUN_L, left precuneus; CERCRU2_L, left crus II of cerebellar hemisphere; CER10_R, right lobule X of cerebellar hemisphere; *represents *p* < 0.05.

**Figure 3:**
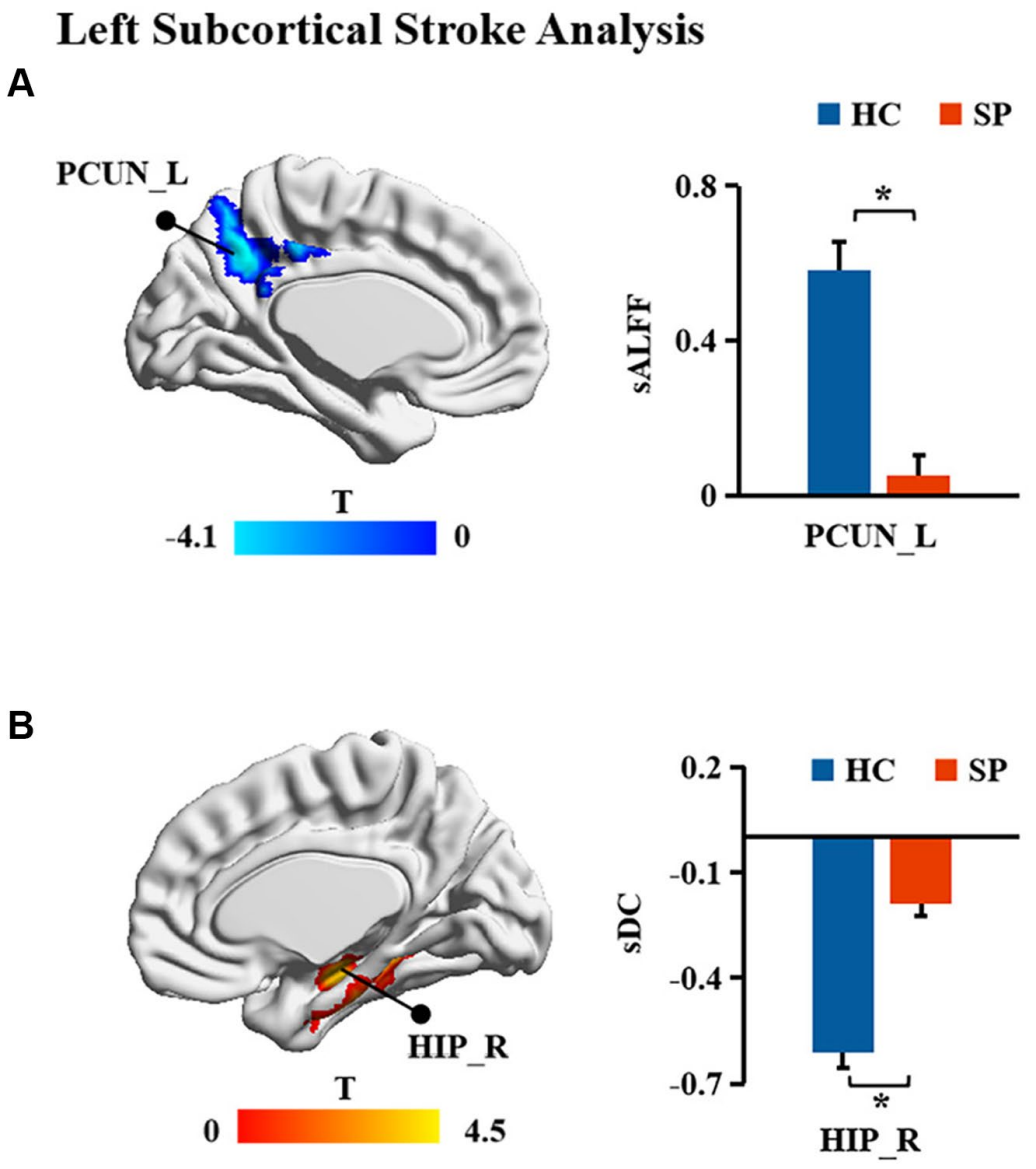
**(A,B)** Brain regions with significant differences in functional measurements between patients with left subcortical stroke compared with health controls (HC). SP, stroke patients; sALFF, static amplitude of low-frequency fluctuation; sDC, static degree centrality; PCUN_L, left precuneus; HIP_R, right hippocampus; *represents *p* < 0.05.

Significant differences in dALFF, sALFF, and sDC between stroke patients and HC were found (Table 2; Figures 2, 3). Significantly decreased dALFF in the left angular (ANG_L) and right inferior parietal gyrus (IPG_R; Table 2; Figure 2A) and decreased sALFF in the left precuneus (PCUN_L; Table 2; Figure 2B) were detected in patients with right subcortical stroke relative to HC. Compared with HC, patients with right subcortical stroke also showed significantly decreased sDC in the left crus II of the cerebellar hemisphere (CERCRU2_L) and IPG_R but increased sDC in the right lobule X of the cerebellar hemisphere (CER10_R; Table 2; Figure 2C). Patients with left subcortical stroke showed significantly decreased sALFF in the left precuneus (PCUN_L; Table 2; Figure 3A) but increased sDC in the right hippocampus (HIP_R) compared to HC (Table 2; Figure 3B). However, neither significant differences were detected in dALFF between patients with left subcortical stroke and HC nor in dDC between patients with right subcortical stroke or left subcortical stroke and HC.

**Table 2:** Brain regions with significant differences in dALFF, sALFF, and sDC between stroke patients and health controls.

Group comparisons	Metrics	Peak label (AAL3)	Number of voxels	Peak MNI coordinate (x, y, z)	Peak T value
Right subcortical stroke analysis	dALFF	Inferior parietal gyrus, R	160	60, −48, 51	−4.0882
		Angular, L	125	−51, −69, 39	−4.05146
	sALFF	Precuneus, L	385	−3, −57, 42	−4.65041
	sDC	Lobule X of cerebellar hemisphere, R	1,074	30, −33, −42	5.47373
		Crus II of cerebellar hemisphere, L	283	−33, −78, −45	−4.67156
		Inferior parietal gyrus, R	213	60, −48, 51	−4.11444
Left subcortical stroke analysis	sALFF	Precuneus, L	200	−6, −57, 39	−4.05113
	sDC	Hippocampus, R	193	36, −18, −18	4.45938

dALFF, dynamic amplitude of low-frequency fluctuation; sALFF, static amplitude of low-frequency fluctuation; sDC, static degree centrality; AAL3, Automated Anatomical Labeling 3 template; R, right; L, left; MNI, Montreal Neurological Institute; Peak T value < 0 indicates stroke patients < health controls.

### Correlation analysis

**Figure 4:**
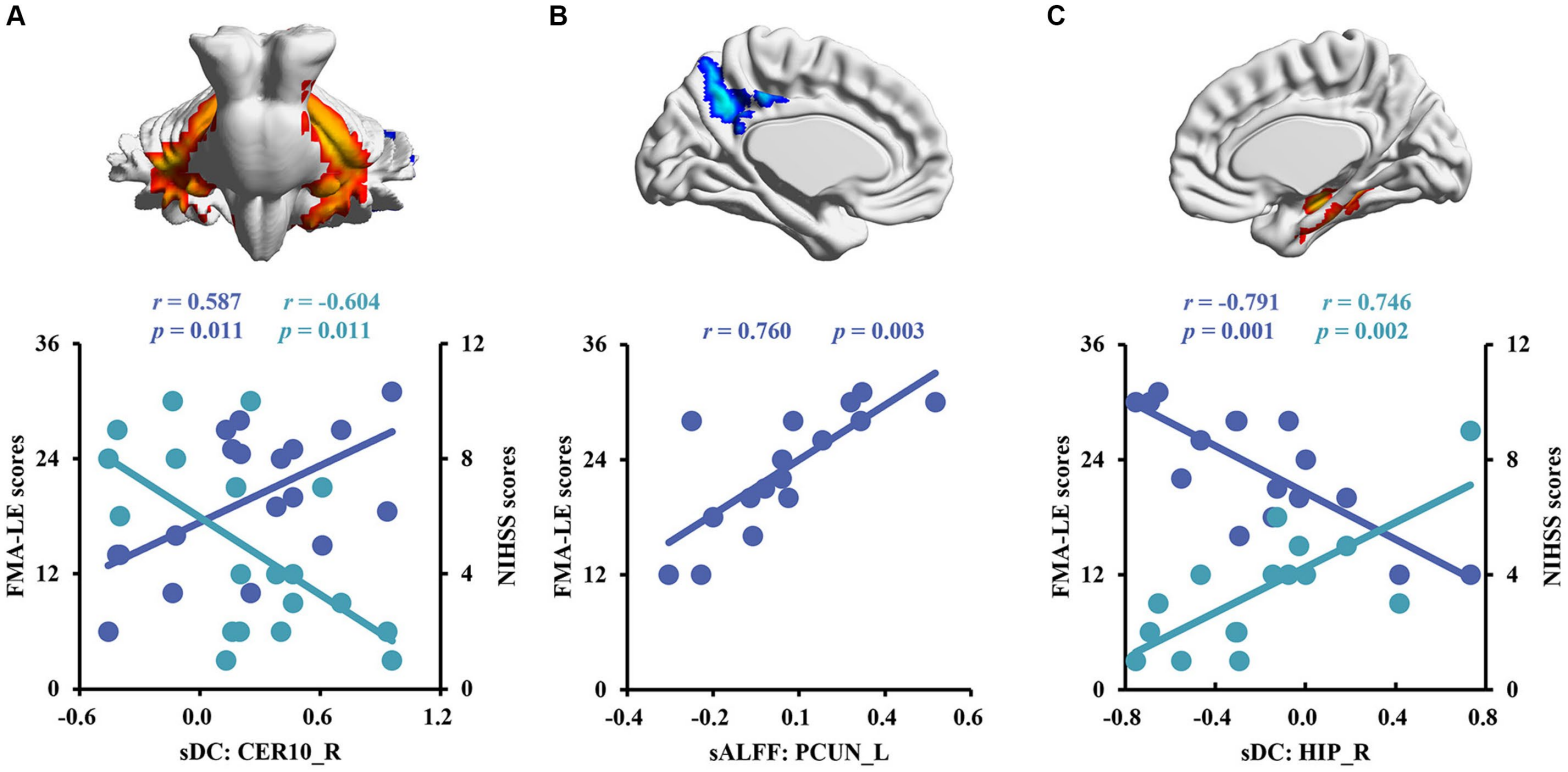
Correlation analysis between brain functional measurements and clinical symptoms of stroke patients. **(A)** The associations between altered sALFF in patients with right subcortical stroke and scores of clinical scales. **(B,C)** The relationships between altered sALFF and sDC in patients with left subcortical and scores of clinical scales. sDC, static degree centrality; sALFF, static amplitude of low-frequency fluctuation; CER10_R, right lobule X of cerebellar hemisphere; PCUN_L, left precuneus; HIP_R, right hippocampus; NHISS, National Institute of Health Stroke Scale; FMA-LE, Fugl–Meyer Lower Extremities assessment; the blue scatters and trend lines indicate FMA-LE scores were correlated with brain functional measurements; the green scatters and trend lines indicate NIHSS scores were correlated with brain functional measurements.

The sDC values in the CER10_R of patients with right subcortical stroke showed a significant negative correlation with NIHSS scores (*r* = −0.604, *p* = 0.011) but a significant positive correlation with FMA-LE scores (*r* = 0.587, *p* = 0.011; Figure 4A). A significant positive correlation was detected between the sALFF values in the PCUN_L of patients with left subcortical stroke and the FMA-LE scores (*r* = 0.760, *p* = 0.003; Figure 4B). In addition, the sDC values in the HIP_R of patients with left subcortical stroke showed a significant positive correlation with NIHSS scores (*r* = 0.746, *p* = 0.002) but a significant negative correlation with FMA-LE scores (*r* = −0.791, *p* = 0.001; Figure 4C).

## Discussion

In the present study, we adopted dynamic and static analysis to investigate brain functional activity and integration in patients with subcortical stroke. Furthermore, we assessed the relationships between brain functional measurement changes and clinical performances of stroke patients. Group comparison results showed that significant alterations of dALFF in ANG_L and IPG_R, sALFF in PCUN_L, and sDC in CERCRU2_L, IPG_R, and CER10_R were found in patients with right subcortical stroke compared with HC. Changes in sALFF of PCUN_L and sDC of HIP_R were observed in patients with left subcortical stroke relative to HC. However, neither significant differences were detected in dALFF between patients with left subcortical stroke and HC nor in dDC between patients with right subcortical stroke or left subcortical stroke and HC. Additionally, the DC values in the CER10_R of patients with right subcortical stroke, sALFF values in the PCUN_L, and DC values in the HIP_R of patients with left subcortical stroke were correlated with the clinical symptoms of patients.

In this study, we found significantly decreased brain functional activity and integration of brain regions in stroke patients mainly located in default mode networks (DMN; Geng et al., 2022), including ANG, IPG, and PCUN. The DMN is a critical resting-state network of the brain and plays an important role in emotional and higher-order cognitive processes (Buckner et al., 2008). It has been confirmed that the disruption of the structure and function of DMN caused by stroke is related to the cognitive deficit of patients (Zhao L. et al., 2018; Wu et al., 2021; Cao et al., 2022). Malouin et al. reported that PCUN and IPG were activated when demands of locomotor tasks require increasing cognitive and sensory information processing (Malouin et al., 2003), suggesting that cognitive-related brain areas contribute to motor function. Zhang et al. found that stroke patients showed significantly increased functional connectivities between the ipsilesional primary motor cortex and the ipsilesional inferior parietal cortex as well as the contralesional ANG (Zhang et al., 2016), which is a possible compensatory mechanism of functional impairments in patients with motor deficit following stroke. Zhao et al. reported changes in functional connectivity of the ipsilesional inferior parietal lobule correlated with the FMA scores (hand and wrist) in patients with stroke (Zhao Z. et al., 2018). In the present study, decreased dALFF in ANG_L and IPG_R were observed in patients with right subcortical stroke compared with HC, suggesting that the dynamics of neural activity in not only the ipsilesional hemisphere but also the contralesional hemisphere are disturbed after right subcortical stroke. Decreased sALFF in the PCUN_L of patients with right subcortical stroke is different from brain regions with altered dALFF, revealing that dynamic brain activity analysis may supply complementary information for static brain activity analysis. Decreased sDC was detected in the IPG_R in patients with right subcortical stroke which suggested that functional integration of ipsilesional IPG was damaged. We also found the sALFF values in the PCUN_L of patients with left subcortical stroke, and a significant positive correlation was detected between those values with FMA-LE scores. This result may provide theoretical basis for lower extremities motor deficit was associated with the disturbance of brain activity in the ipsilesional PCUN after the left subcortical stroke. Although abnormal brain functional activities and integration were found in cognitive-related brain regions, they were not associated with cognitive deficit of patients. Possible reasons may be because of the cognitive deficit of patients and the sample size of stroke patients is relatively small.

In addition, we also found decreased sDC in the CERCRU2_L while increased sDC in CER10_R in patients with right subcortical stroke relative to HC. The sDC values in the CER10_R are closely related to the severity of the stroke and the lower extremities motor function of patients. The cerebellum is involved in motor, coordination, and balance control functions (Ouchi et al., 1999; Aoki et al., 2005; Barmack and Pettorossi, 2021) as well as non-motor functions, such as emotion, executive function, language, music, verbal working memory, and internal timing (Keren-Happuch et al., 2014). A previous resting-state fMRI study of healthy young adults revealed high functional connectivities between CERCRU2 and the supplementary motor area (SMA), thalamus, caudate nucleus, and hippocampus, as well as between CER10 and SMA, DMN, and HIP (Sang et al., 2012), supporting the important roles of CERCRU2 and CER10 in complex movements and cognition. Liu et al. reported a significant correlation between the DC values of the ipsilesional CERCRU1 and CERCRU2 and FMA scores in patients with pontine infarction (Liu et al., 2019). Our results revealed that functional integration of the cerebellar network was disrupted in patients with right subcortical stroke. The increased sDC in CER10_R may be the result of functional brain network reorganization in the early stage of stroke, which benefits the lower extremities motor function of stroke patients.

Finally, we found increased sDC in the HIP_R of patients with left subcortical stroke compared to HC. HIP is strongly associated with learning and memory. Patients suffer from memory deficits when the lesion of stroke involves HIP (Szabo et al., 2009). However, few studies reported the correlation between the structure and function of HIP and motor function of stroke patients. A 1-year longitudinal study by Fan et al. showed that the gray matter volume of HIP was related to the motor index of patients with left subcortical infarction (Fan et al., 2013), which provides evidence that the structural reorganization of HIP may be beneficial for motor recovery. Another half-year longitudinal study by Shan et al. reported that increased voxel-mirrored homotopic connectivity (VMHC) was detected in the HIP/amygdala within 7 days after stroke, and this value was maintained at a higher level in the next four time points. In addition, the VMHC values in HIP/amygdala were negatively correlated with FMA scores at all time points (Shan et al., 2018). In our study, the sDC values in the HIP_R of patients with left subcortical stroke showed a positive correlation with NIHSS scores but a negative correlation with FMA-LE scores. Our results may provide a theoretical basis for the association between HIP and the severity of the stroke and motor function deficit of stroke patients at the level of brain network analysis.

This study has some limitations. First, the sample size of stroke patients is relatively small, which may lead to unstable results. Given that our results are corrected with multiple comparisons correction, these results are reliable. Second, the inclusion criteria were stroke patients with motor impairment, but our results did not find changes in brain functional activities or integration in motor-related brain regions, such as the primary motor area, premotor cortex area, and supplementary motor area. This may be owing to the heterogeneity of stroke lesions and the varying degrees of motor deficit of patients. Categorizing patients into subgroups according to the degree of motor deficit may get better results. Third, our results found changes in functional measurements of cognitive-related brain regions. However, we did not find any measurement correlation with cognitive deficits. Possible reasons may be because of the cognitive deficit of patients and the sample size of stroke patients is relatively small. Studies with large sample sizes are needed to further explain this association.

In conclusion, abnormal brain functional activities and integration were found in the hippocampus, default mode network, and cerebellar network of patients, implying a simultaneous change of multiple brain networks following stroke. Patients with right and left subcortical stroke showed different change patterns. In addition, brain regions with altered dALFF diverged from those with altered sALFF after right subcortical stroke, which may suggest that time-varying brain activity analysis provides complementary information for static brain activity analysis. Dynamic and static brain functional activity and integration analysis may contribute to a more comprehensive understanding of the underlying neuropathology of dysfunction in stroke patients.

## Data availability statement

The original contributions presented in the study are included in the article/supplementary material, further inquiries can be directed to the corresponding authors.

## Ethics statement

The studies involving humans were approved by the Ethics Committee of the Affiliated Hospital of Yunnan University. The studies were conducted in accordance with the local legislation and institutional requirements. The participants provided their written informed consent to participate in this study. Written informed consent was obtained from the individual(s) for the publication of any potentially identifiable images or data included in this article.

## Author contributions

JW, DQ, and YY designed this study. LG, ZZ, and XY participated into the participants recruitment and acquired the clinical assessments data. LG, ZZ, and PW acquired the MRI data. LG, WS, and JW analyzed the MRI data. LG wrote the manuscript’s first draft. ZZ, XY, PW, JW, DQ, and YY revised the manuscript and provided the critical comments. All authors contributed to the article and approved the submitted version.

## Footnotes

^1^ http://itksnap.org

^2^ https://www.graphpad.com
